# Addressing the Disproportionate Impact of COVID-19 on Communities of Color

**DOI:** 10.1007/s40615-021-00989-7

**Published:** 2021-03-19

**Authors:** Garth Graham

**Affiliations:** CVS Health, Woonsocket, RI USA

**Keywords:** COVID-19, Pandemic, Community-based testing, Minority populations, Racial inequities, CVS Health, COVID-19 testing, Telehealth, MinuteClinic, Chronic diseases, Health care, Underserved communities

## Abstract

With Black and Hispanic communities across the USA experiencing more detrimental negative effects from the COVID-19 pandemic as compared with other demographic groups, the virus has exposed the racial and ethnic disparities in treatment and care that public health experts have been grappling with for years. This paper explains how the systematic collection of racial and ethnic data gleaned from COVID-19 testing in underserved communities can be used to better understand this pandemic and inform measures within our control to prevent the spread of disease in the future.

The detrimental impact of COVID-19 continues to be seen and felt across the USA. However, with Black and Hispanic communities experiencing more significant negative effects from the pandemic as compared with other demographic groups, the virus has exposed the racial and ethnic disparities in treatment and care that public health experts have grappled with for years.

When COVID-19 case numbers increase among our minority populations, so too does the data demonstrating the systemic racial inequities that have long plagued our health care system, as members of these groups struggle to access and afford health care. We have seen these historically pervasive inequities magnified in real time through the spread of COVID-19, with a recent analysis showing that US counties with a majority share of Black or Hispanic residents have case rates and death rates above the national average. Black and Hispanic communities also face greater financial impacts, higher rates of infection, and higher rates of death. And, when age is taken into account, the death rate for Black and Hispanic Americans is 3.6% and 2.5%, respectively, times that of Whites, according to recent research from the Brookings Institution.

By working together to expand access to COVID-19 community-based testing, elected leaders, public health officials, and the business community have an unprecedented opportunity to prevent the continued spread of COVID-19 in at-risk communities *and* make longer-term changes to eliminate health disparities among minority and underserved populations. The systematic collection of racial and ethnic data gleaned from COVID-19 testing in underserved communities can be used to better understand this pandemic and inform measures within our control to prevent the spread of disease in the future. The testing data can also enable us to better understand and address the country’s longstanding and pervasive health care disparities, by providing evidence and support for a more equitable allocation of resources and funds based on the specific needs of certain demographics.

## Rapidly Advance Testing in Low-Income Communities

Quelling the impact of COVID-19 among minority populations begins with doing more to reach into minority communities to provide expanded testing and other resources. Tailored to the needs and culture of the local population, community-based testing allows us to reach individuals in highly vulnerable and disadvantaged populations who may not otherwise have access to testing because of racial disparities like socio-economic status, living conditions, and language or educational barriers.

It is critical that access to COVID-19 testing be expanded to the underserved, multicultural communities that are being disproportionately impacted by the pandemic. That is why CVS Health has built additional testing infrastructure in local communities, particularly those hit hardest by the pandemic, to bring testing resources to uninsured and underserved individuals.

CVS Health currently manages the largest number of independently run COVID-19 test sites in the country, and in collaboration with the US Department of Health and Human Services, we’ve launched numerous community-based testing sites in underserved communities across the country.

We have more than 3,000 CVS Pharmacy locations in 33 states and the District of Columbia offering no-cost self-swab testing, with more than 4,000 COVID-19 testing sites operational across the country. More than half of the test sites we have established serve counties with communities with demonstrated need for support, as measured by the Centers for Disease Control and Prevention’s Social Vulnerability Index. The index tracks a variety of census variables including poverty, lack of access to transportation, and crowded housing that may weaken a community’s ability to prepare for and recover from hazardous events like natural disasters and disease outbreaks.

## Mobilize Community Organizations

Alliances between health care organizations and local groups are critical to effectively increasing access to COVID-19 testing in underserved populations that are being hit the hardest by health inequities. Working with our community organizations and accommodating walk-up testing is an important part of our comprehensive strategy to increase access to COVID-19 testing. To do this, CVS Health has continued to work with a number of community organizations across the country, including free clinics, community colleges, and faith-based institutions, to open rapid-response community testing sites that aim to increase access to testing for uninsured and underserved populations. By co-locating testing sites at outside organizations’ facilities, CVS Health has been able to tap into local networks to target and expand testing in areas that have the greatest need.

Our rapid-result COVID-19, community-based testing sites are helping increase access to testing for more people impacted by the pandemic, particularly groups that have existing challenges with access to care. These sites can accommodate walk-up testing, so access to a vehicle is not required and appointments can be made by phone, so lack of internet access is not a barrier.

Additionally, recognizing that patients from diverse populations are more trusting of doctors from their own neighborhood, expanding community-based testing in underserved areas can also help to encourage those who may be distrustful of the health care system and more prone to delay care. CVS Health has prioritized collaborating with organizations serving various minority populations to increase awareness of testing options in their area and make sure there is equitable access to testing, including the National Medical Association (NMA). As the nation’s oldest and largest organization of Black physicians, collaborations like these can help drive more Black community members to rapid COVID-19 testing sites and allow medical professionals to engage with underserved groups in these communities.

As we potentially expand testing sites to other locations, CVS Health will use a similar strategy with NMA local chapters. We also will engage community organizations that we and our affiliated charitable foundations have developed relationships with over many years—such as members of the National Association of Free and Charitable Clinics—to raise greater awareness of COVID-19 symptoms and access to testing. Working with groups that have strong community ties has allowed CVS Health to further expand testing in areas of greatest need. Not only are we increasing access for people who might otherwise go without medical diagnosis, but we are also gaining a better understanding of the spread of COVID-19 within vulnerable populations and throughout the country.

## Launch Targeted Specific Health Messages Aimed at Preventive Actions

Community organizations are also critical allies in raising greater awareness of COVID-19 symptoms and access to testing. While it is an uncertain time for our nation’s health, and we’re only beginning to see the ripple effects of gaps in treatment for chronic diseases, our aim is always to care for the patients we serve, especially the most vulnerable.

That’s why CVS Health is trying to help educate all Americans about the importance of maintaining treatment for chronic disease, dispel fears preventing people from going to the hospital or doctor’s office, and provide key resources and tools for patients and health care providers alike. The NMA and other community-focused organizations can serve as trusted resources and reinforce the importance of measures that all individuals can take to help prevent the spread of the virus, such as social distancing and proper handwashing.

## Engage Health Care Providers

Another negative consequence of the pandemic has been that many people with chronic conditions are delaying care due to fear of contracting COVID-19 in a health care setting. It is well-documented that members of underserved communities face higher rates of conditions like diabetes, high blood pressure, and heart disease. The Centers for Disease Control and Prevention reports that young Black Americans are dealing with diseases more common at older ages, such as high blood pressure, diabetes, and stroke. Hispanic and Latino Americans have a 50% chance of developing type 2 diabetes. While much is still unknown about COVID-19, the impacts of these devastating conditions are well-known, and treatment and preventive care protocols are well-established. It is absolutely critical for those suffering or at risk to continue regular doctor visits and seek treatment in the event of a medical emergency such as a stroke or heart attack.

Health care professionals across the country, but especially those located within minority communities, need to understand this issue and make themselves more readily available for health issues not related to COVID-19, while at the same time creating protocols to keep their patients safe. For example, Aetna, a CVS Health company, has been proactively reaching out to members with chronic conditions who may be at higher risk for COVID-19 and providing support to not only keep them from contracting the disease, but also to make sure they are continuing to receive the care they need for their existing health issues. Telehealth has experienced a huge surge in adoption over the course of the pandemic, allowing patients and providers to connect virtually without risk of exposure to the virus. In fact, the utilization of telemedicine for virtual visits through our MinuteClinic locations have been up significantly since the pandemic began.

Yet, even though the ability to see patients virtually can be a huge advantage in helping limit the spread of COVID-19, there are certain health issues that benefit from being addressed in a person-to-person setting. Making sure that people know it is safe to receive care in a clinical setting if it is needed is essential to maintaining the overall health of the population, particularly for individuals with chronic diseases.

